# Deep reinforcement learning and fuzzy logic controller codesign for energy management of hydrogen fuel cell powered electric vehicles

**DOI:** 10.1038/s41598-024-81769-1

**Published:** 2024-12-28

**Authors:** Seyed Mehdi Rakhtala Rostami, Zeyad Al-Shibaany, Peter Kay, Hamid Reza Karimi

**Affiliations:** 1 School of Engineering, University of the West of England Bristol, Bristol, UK; 2 Computer Engineering Department, University of Technology, Baghdad, Iraq; 3 The Engineering and Design Institute, London, UK; 4 Department of Mechanical Engineering, Politecnico di Milano, Milan, Italy

**Keywords:** Fuel cell electric vehicle, PEM fuel cell, Ultracapacitor, Deep reinforcement learning, Fuzzy control, Energy management, Electrical and electronic engineering, Fuel cells

## Abstract

Hydrogen-based electric vehicles such as Fuel Cell Electric Vehicles (FCHEVs) play an important role in producing zero carbon emissions and in reducing the pressure from the fuel economy crisis, simultaneously. This paper aims to address the energy management design for various performance metrics, such as power tracking and system accuracy, fuel cell lifetime, battery lifetime, and reduction of transient and peak current on Polymer Electrolyte Membrane Fuel Cell (PEMFC) and Li-ion batteries. The proposed algorithm includes a combination of reinforcement learning algorithms in low-level control loops and high-level supervisory control based on fuzzy logic load sharing, which is implemented in the system under consideration. More specifically, this research paper establishes a power system model with three DC-DC converters, which includes a hierarchical energy management framework employed in a two-layer control strategy. Three loop control strategies for hybrid electric vehicles based on reinforcement learning are designed in the low-level layer control strategy. The Deep Deterministic Policy Gradient with Twin Delayed (DDPG TD3) is used with a network. Three DRL controllers are designed using the hierarchical energy optimization control architecture. The comparative results between the two strategies, Deep Reinforcement Learning and Fuzzy logic supervisory control (DRL-F) and Super-Twisting algorithm and Fuzzy logic supervisory control (STW-F) under the EUDC driving cycle indicate that the proposed model DRL-F can ensure the Root Mean Square Error (RMSE) reduction for 21.05% compared to the STW-F and the Mean Error reduction for 8.31% compared to the STW-F method. The results demonstrate a more robust, accurate and precise system alongside uncertainties and disturbances in the Energy Management System (EMS) of FCHEV based on an advanced learning method.

## Introduction

Fc electric hybrid vehicles have become a hot topic today as they have the potential to improve zero-emission, aiming to mitigate environmental degradation, and improve the fuel economy and performance of power sources in real-time^1^. Transportation is one of the key contributors to global warming and air pollution. Then, there has been a growing demand for electrified vehicles that utilize electric motors in their powertrain and, thus, emit fewer pollutants. Fossil fuel cars and vehicles will be swapped for new electric vehicles in the future. Because of its safety, cost-effectiveness, and other advantages, lithium-ion batteries are widely used in electric vehicles^2,3^. Despite the high energy density of the Li-ion batteries, their power densities are not high enough for EV applications, making them a low-efficiency element for the rapid exchange of energy. Because it is beneficial to combine batteries with another complementary storage resource, such as UC, that provides high power density and low energy density^4^. To make the current transportation system more environmentally friendly, a variety of vehicle electrification technologies, including FCHEVs, plug-in hybrid electric vehicles (PHEVs), hybrid electric vehicles (HEVs), and battery electric vehicles (BEVs), have been developed. FCHEVs are showing a consistent increase in the market segment for road cars, to the point where numerous prototypes of various brands and sizes, such as the Hyundai Nexo, Honda Clarity, Mercedes-Benz F-Cell, and Toyota Mirai, have been produced^5–9^.

The PEMFC, with its solid electrolyte, high power density, and low operating temperature, has the most potential of all the FC types to be used in automotive applications. Table 1 presents a comparison of various electric cars. Taking advantage of the high energy density and short refuelling time. However, the higher cost and short life of the PEMFC system and battery in an electric vehicle diminish the fuel cell electric vehicle from becoming the main transportation solution^10^.

**Table 1:** Markets, companies and FCHEV^6–9^.

Type of vehicles	Markets and companies	Strength	Weakness
PHEV (ICE + Bat)	Mercedes-GLC360e	• Less emission than HEV • Convenient charging	• Complex powertrain • Higher battery cost
	Honda-Clarity		
	Audi A3 E-Tron 2016		
BEV (Only Bat)	Tesla-Model S	• Zero exhaust emission • Convenient charging • Smooth propelling without noise	• Long recharging time • Shorter battery lifetime rather than hybrid
	Nissan-Leaf^6^		
	Kia-Soul		
FCHEV (FC + Bat + and/or UC)	Toyota-Mirai^7^	• Fast refuelling • Smooth propelling without noise • High driving autonomy	• Immature technology • Restriction for infrastructures • High cost
	Hyundai ix35 FUEL CELL^8^		
	Kia Borrego FCEV^9^		

However, a PEMFC includes some restrictions, such as its slow dynamic and inability to store energy, which prevent it from meeting all the needs of vehicles. Due to this, using a secondary power source such as a battery or UC is required to meet the fast-dynamic load in automobiles, lower the rate of PEMFC degradation by absorbing power peaks, improve fuel efficiency, power the load during cold start, and recover energy. Three common configurations for hydrogen vehicle hybridization are FC-battery, FC-UC, and FC-battery-UC^11^. The battery and the supercapacitor serve as auxiliary sources, whereas the FC serves as the primary source in the Hybrid Energy Storage System (HESS) paradigm, which has one main source and two energy storage sources. There are multiple possible configuration types of an FCHEV listed in Table 2^12^.

**Table 2:** Multiple possible configurations.

	PEMFC	Battery and/or SC	
Connection (DC/DC converter)	YES	YES	Full-active
	YES	NO	Semi-active
	NO	NO	Passive
	NO	YES	Not common

### Control issues on energy management strategies

#### Rule-based/fuzzy logic control energy management strategies

In the rule-based energy management strategy, a set of predefined rules or look-up tables determine power sharing.

The main benefits of the fuzzy rule-based strategy in the EMS of HESS are low computational load, robustness, easy implementation, and high reliability^13^. They are cost-effective nonlinear control and intelligent and smart. The fuzzy controller output has good smoothness. And it is dependent on the fuzzy membership functions and rules^13^.

**Fig. 1:**
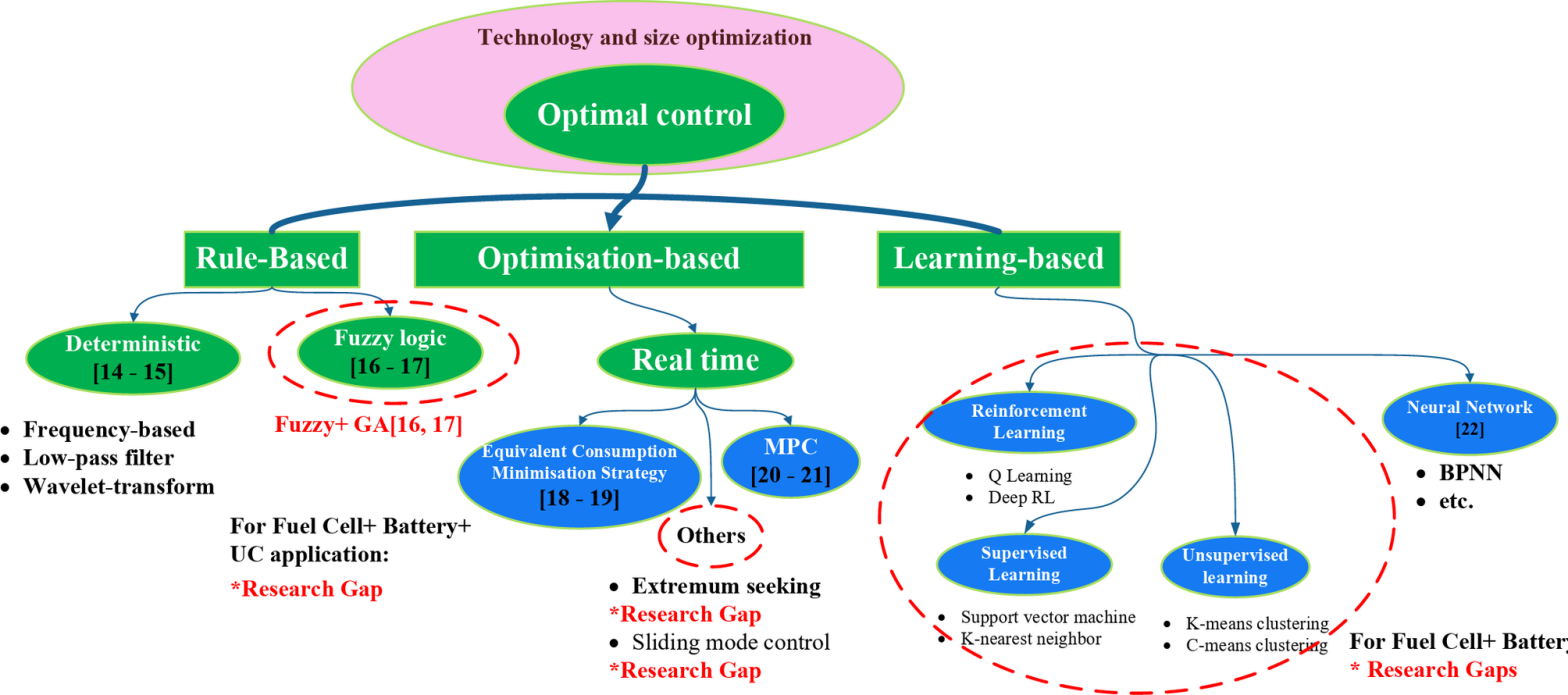
Review of different energy management systems in Fuel cell + battery + UC Electric vehicle system.

The main disadvantage of the fuzzy rule-based strategy in the EMS of HESS is that fuzzy controller design depends on the engineer’s experience and knowledge. A summary of optimization-based Energy management systems in FCHEVs based on Fuel cell, battery, and UC are presented in Fig. 1 and Table 3. In several classifications in the literature, recent energy management systems have been found in research and classified into three methods: Rule-based, optimization-based, and learning-based, as shown in Fig. 1 and Table 3.

**Table 3:** Classification of different energy management systems in Fuel cell + battery + UC Electric vehicle system.

Proposed method	Vehicle type	Objective function	Management system performance description
Equivalent Consumption Minimisation (ECMS) + Fuzzy	Mining truck (FC + BAT + UC)	Minimize the hydrogen consumption	Fuel consumption was reduced by 7.94% using an ECMS with a fuzzy controller^23^
State machine control + ECMS	Tram (FC + BAT + UC)	Maximize the overall efficiency including distance, speed, fuel economy and FCS efficiency	Better fuel economy decreased by 3.2% and stack efficiency increased by 6.74%^24^
Meta-model based global optimization algorithm	Mining truck (FC + BAT + UC)	Minimize the life cycle cost	Minimize the overall operation costs of FCEVs, including the cost of hydrogen fuel, the cost associated with the degradations of the PEMFC system^25^
Dynamic Programming (DP)	Transit bus (FC + BAT + UC)	Minimize the percentage deviation of the hydrogen consumption and the average absolute battery power	DP consumes the same amount of hydrogen with a deviation of less than 0.05%^26^
Efficient convex programming	Others (FC + BAT + UC)	Minimize the percentage deviation of the hydrogen consumption	Reduce the average absolute battery power^27^
Rule-based and dynamic programming (DP)-based	Others (FC + BAT + UC)	Improves the fuel economy and system durability of vehicles and hydrogen consumption	FCs struggles to fulfil the vehicle’s constant fluctuating power requirement under real-world driving situations^28^
Deep reinforcement learning-based energy management for cabin comfort control	Fuel cell vehicles (FCVs) without UC	To optimize powertrain energy consumption	Comprehensive control of cabin comfort and fuel cell/battery durability is proposed to increase energy efficiency of FC bus^29^
Deep RL energy management for fuel cell buses based on TD3 algorithm	Fuel cell Buses, (FC + BAT)	Fuel cell durability, and Li-ion Battery thermal health awareness	TD3-based energy management system can improve battery life by 28.02% and overall vehicle economy by 8.92%^30^
Bi-directional long short-term memory for degradation prediction model for PEMFC	Just Fuel cell system	Fuel cell durability, lifetime prediction	Proposed BiLSTM model achieves accurate predictions under different current conditions^31^

## Main contribution

The contributions of this research manuscript are summarised as follows:

1. An energy management system including Deep Reinforcement Learning (DRL) and fuzzy logic control-based load sharing is implemented for the dynamic PEMFC/Battery/UC hybrid vehicular power system model. A hierarchical energy management framework is employed in a two-layer control strategy. In the low-level layer control strategy of the hierarchical energy management structure, three loop control strategies for hybrid electric vehicles based on reinforcement learning are designed to improve FC and battery power-sharing tracking and voltage bus.
2. The Twin Delayed Deep Deterministic Policy Gradient (TD3) approach is used as one of the advanced DRL methods to develop an intelligent energy management strategy in low-level loops (Bus line voltage tracking loop—Fuel cell power tracking loop, and battery power tracking loop) for FCHEV. For the complex continuous state-action space of FCHEV, a DDPG-based algorithm is introduced, and a hierarchical energy management framework is employed to reduce the dimensionality of the action-state space.
3. In this work, the Deep Deterministic Policy Gradient with Twin Delayed (DDPG TD3) was used with a network. Three RL-PID controllers are developed using an artificial intelligence algorithm based on reinforcement learning. This algorithm uses an actor-critic agent whose objective is to optimize the actor’s policy and train a critic for rewards. This will generate the appropriate gains without the need to know the system.
4. Furthermore, fuzzy logic controller in the high-level supervisory control layer offers the following benefits: Simple understanding and easy implementation—Real-time operation- Robustness and adaptability against uncertainty, noise, disturbances and stability handling in various performing situations. Therefore, the proposed method will suggest a more robust, accurate, and precise system alongside uncertainties and disturbances in the EMS of FCHEVs.

However, the engineering process of obtaining a mathematical system modelling often demands in-depth, physical concepts. If some initial conditions change (such as FC temperature, flow rate, FC voltage, Battery voltage, UC voltage, battery SoC and UC SoC, and …), the engineering process must be repeated to obtain an appropriate controller. Conversely, with the DDPG-TD3 training policy, the system continually adjusts itself, eliminating the need for the user to engage in re-engineering for applied control.

This paper is organized as follows: section “Main contribution” introduces a literature review of the paper, and the hybrid topologies used in this study. In section “Topology and structure of HESS”, the topology and structure of HESS is presented and discussed. Modelling of the hybrid energy storage system is presented in section “Modelling of PEMFC, UC, and Li-ion battery”. In section “Energy management strategy three RL loops and high level supervisory fuzzy control”, a proposed energy management strategy with three RL Loops and high-level supervisory fuzzy control is developed. The evaluations, results and analysis are performed in section “Evaluations and results”. Finally, conclusions drawn from the study are given in section “Conclusion”.

## Topology and structure of HESS

**Fig. 2:**
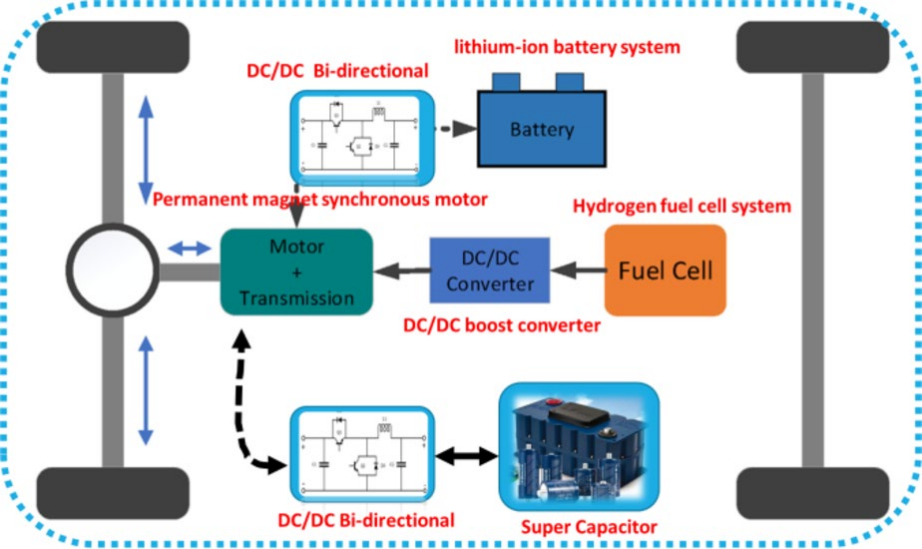
Structure of Hydrogen FC – Battery − UC on fuel cell hybrid electric vehicle.

A typical FCHEV powertrain design is shown in Fig. 2. An FCHEV is a car with these kinds of structures, where the primary energy source is a PEMFC stack, and the secondary source is either a Li-ion battery pack or a UC bank. In this framework, a PEM fuel cell, lithium-ion battery bank, and Ultra-capacitor establish a connection to the DC link. The HESS should have high power density, extended working life, and relatively high efficiency. Therefore, HESS consists of a FC connected with a boost converter, the lithium-ion battery bank and the Ultra-capacitor to maintain a link to the DC bus via two bidirectional DC-DC converters. The DC bus, on the other side, is connected to a DC-AC inverter used to drive the vehicle’s traction motor. The inverter plays a critical role, delivering DC power to its AC counterpart, thereby empowering the motor to actuate the vehicle’s transmission system. The traction system consists of a combination of power inverters and electric motors that are expertly coordinated by control systems.

### Full-active topology for PEMFC-Li-ion battery-UC

**Fig. 3:**
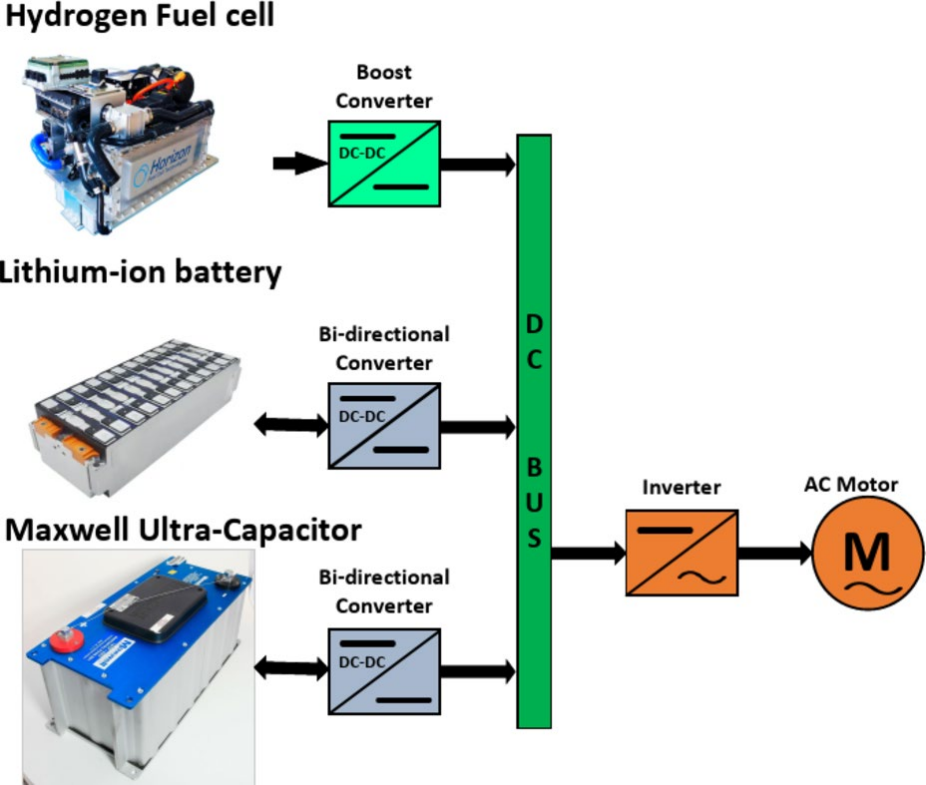
Full-active HESS topology including the hydrogen FC, Li-ion battery, and UC.

A full-active HESS topology, including the PEMFC and Li-ion battery and Ultra-capacitor, is illustrated in Fig. 3. This full-active HESS type has significant controllability and enables customized power allocations. This facilitates efficient energy management and the best possible use of every energy storage resource according to its unique charge/discharge properties.

**Fig. 4:**
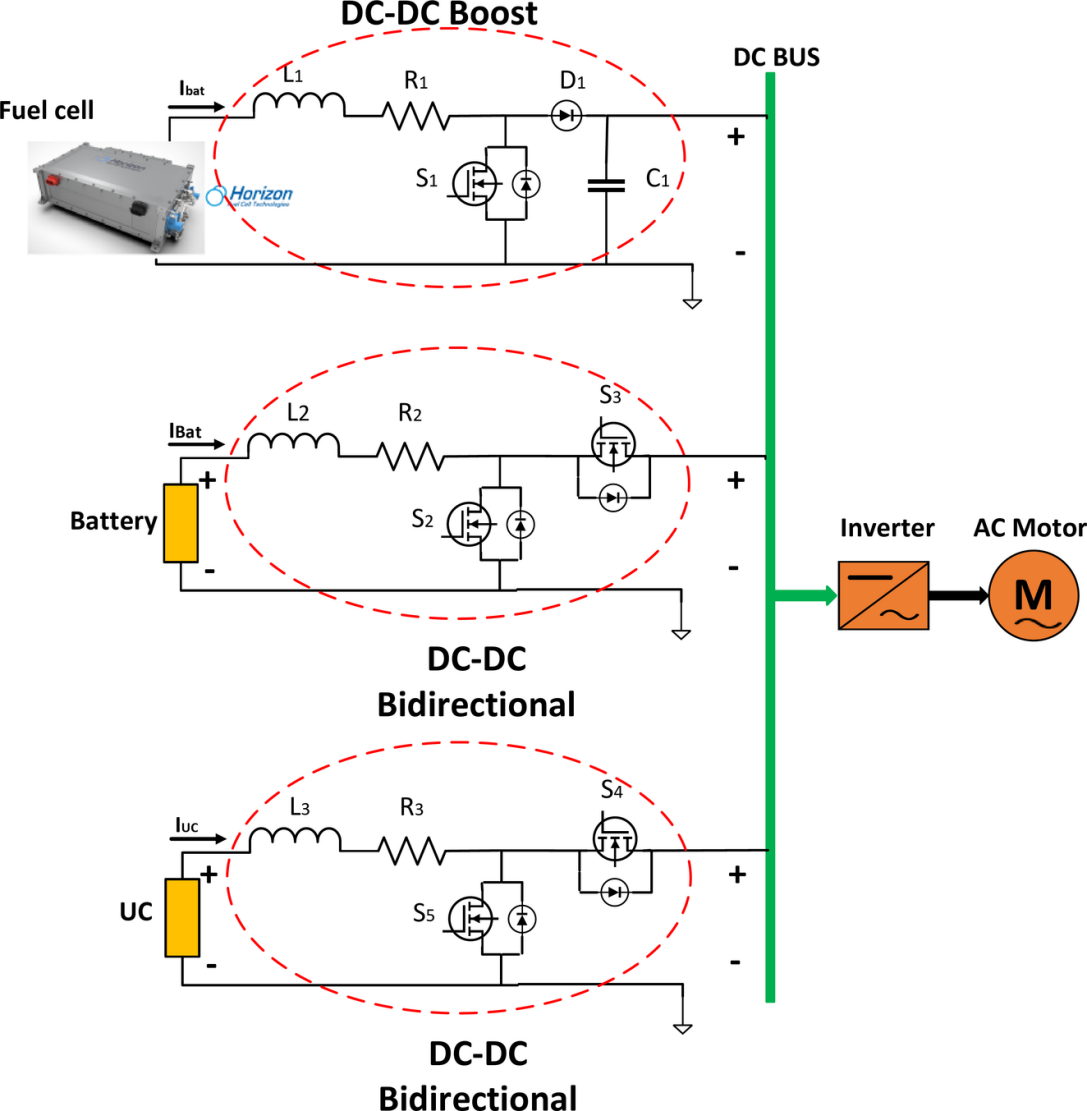
Circuit diagram of HESS in FCHEV.

Figure 4 details the battery, Ultracapacitor, FC, and load and DC-DC converters. There are two non-isolated bidirectional DC-DC converters and one DC- DC boost converter in full-active HESS topology that are connected by power switches *S*_1_, *S*_2_, *S*_3_, *S*_4_, and *S*_5_. The detailed circuit topology is shown in Fig. 4.

For discharge mode of FC, battery, and UC:

- Switches *S*_1_ and *S*_2_ are engaged, thus being in the on state, whereas switch *S*_3_ remains off. Here, diode *D*_*0*_ and the antiparallel diode *D*_3_ assume the roles of freewheel diodes, facilitating a particular directionality of the current flow.

However, when the load transitions into the discharge mode:

- Switch *S*_3_ becomes activated, shifting into the on state, whilst the switches *S*_1_ and *S*_2_ are deactivated, hence assuming the off state. To maintain a consistent current flow, the antiparallel diodes *D*_1_ and *D*_2_ come into play. Additional components within this circuit include inductive elements denoted as *L*_1_ and *L*_2_, coupled with series equivalent internal resistances represented by *R*_1_ and *R*_2_, contributing to the functioning of the system.

## Modelling of PEMFC, UC, and Li-ion battery

### PEM fuel cell model

The open circuit voltage of a cell is1.229 V. The actual cell potential is decreased because of irreversible losses. The irreversible losses can arise from multiple sources. Three different processes can cause the losses, commonly referred to as polarization over voltage: concentration polarization, ohmic polarization, and activation polarization^32–35^. Symbols used in this paper are tabulated in Table 4.

**Table 4:** Fuel cell voltage parameters.

$N$	Number of cells
$T$	Temperature of fuel cell
$P_{{H_{2} }} ,\; P_{{O_{2} }}$	Partial pressure of each gas inside
$R$	Universal gas constant
$F$	Faraday’s constant
$\alpha$	Charge transfer coefficient
$i$	Output current density
$i_{0}$	Exchange current density
$I_{fc}$	Output current
$r$	Area-specific resistance
*m* and *n*	Constants in the mass transfer voltage

A cell voltage with losses is mentioned in Eq. (1):

1 $$V_{FC} = E - losses$$

Nernst voltage equation is presented form as^32–35^:

2 $$E = 1.229 - 0.85 \times 10^{ - 3} \times \left( {T - 298.15} \right) + 4.3085 \times 10^{ - 5} T \times \left( {\ln \;P_{{H_{2} }} + \frac{1}{2}\ln P_{O2} } \right)$$

The voltage losses due to activation, ohmic and concentration losses are defined in the following:

#### Ohmic loss

The ohmic resistance of the PEM fuel cell includes the resistance of the anode and cathode in electrode manufacturing and the resistance of the polymer electrolyte membrane to the movement of ions. Ohmic voltage loss for a single PEM fuel cell stack can be given as:

3 $$V_{ohmic} = N\; I_{fc } \;r$$

#### Activation loss

This loss is caused by the slowness of the reactions taking place on the surface of the electrodes^32–35^. Dominant at low current density (i.e. at the beginning of V–I characteristic curve).

4 $$V_{act} = N\frac{R T}{{2 \alpha F}} \ln \left( {\frac{i}{{i_{o} }}} \right)$$

#### Concentration loss

This voltage drop results from the change in concentration of the reactants at the surface of the electrodes as the fuel is used. The concentration losses for a single cell stack can be given as^32–35^:

5 $$V_{conc} = N \;m\;\exp (n I_{fc} )$$

Significant at higher currents (at the end of V–I characteristics of the PEM fuel cell).

Hence, the actual output voltage of PEM fuel cell at normal operating conditions^32–35^:

6 $$V_{Cell} = E - V_{ohmic} - V_{act} - V_{conc}$$

*E* is the Nernst instantaneous voltage. A fuel cell stack consists of several cells in series to increase the voltage from fuel cell. In the following equation, $N_{cell}$ is the number of cells in series. Fuel cell stack voltage was described by:

7 $$V_{stack} = N_{cell} \cdot V_{Cell}$$

The PEMFC is proposed in this research that presented in Table 4.

The design parameters used for the simulation purposes have been presented in^37^.

**Fig. 5:**
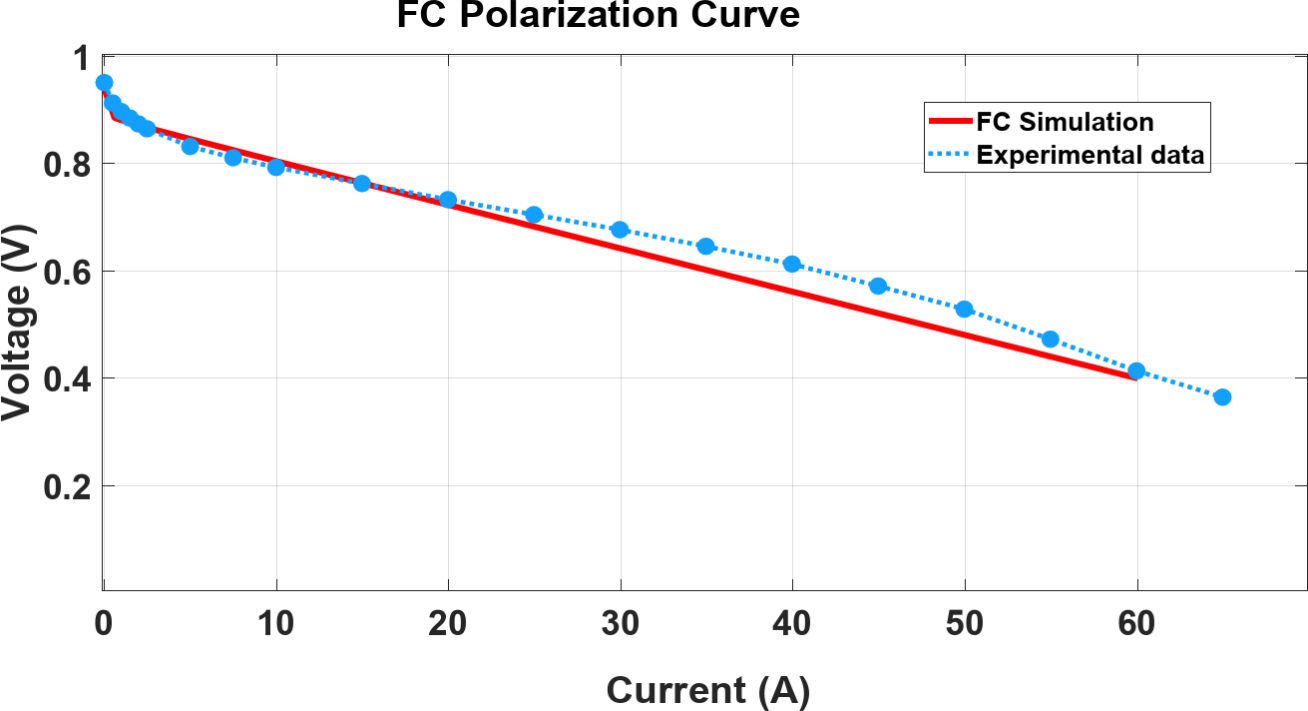
Model verification: Fuel cell polarization curve and experimental evaluation and datasets^36^.

The polarization curve test is one of the key testing techniques for fuel cells to check simulation modelling and experimental test results. Figure 5 shows the polarization curve measured directly after the dynamic load cycle. The complete shared dataset is available on the Mendeley data repository^36^. This figure shows that the developed and used simulated model fits exactly with experimental tests and confirms the simulation model used in FCHEV (Table 5).

**Table 5:** PEMFC specifications^37^.

Parameter	Value
Maximal stack power (W)	35 kW
Nominal voltage (V)	350 V
Nominal current (A)	250 A
Number of cells	400

### Battery model

**Fig. 6:**
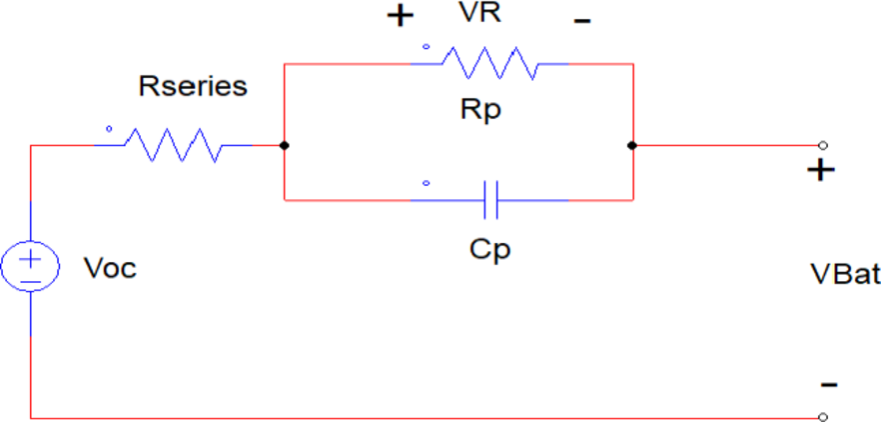
Equivalent Thevenin battery model^5^.

Kirchhoff voltage law and Kirchhoff current law can be used to obtain the state-space equation of the Thevenin model, as shown in Eq. (8). Figure 6^3^ displays the corresponding Thevenin-based electrical model of the Li-ion battery cells utilized in this article. The following equations show the Kirchhoff voltage law and Kirchhoff current law. Equation (8) can be used to obtain the state-space equation.

8 $$\begin{aligned} & \dot{V}_{cp} = \frac{{I_{Bat} }}{{C_{p} }} - \frac{{V_{cp} }}{{R_{p } C_{p} }} \\ & V_{Bat} = V_{ocv}^{Bat} - R_{p} \cdot C_{p } \cdot \frac{{dV_{cp} }}{dt} - I_{Bat} \cdot R_{series } \\ \end{aligned}$$

The Li-ion battery pack output voltage is shown in Fig. 5, and the Li-ion battery pack characteristics are listed in Table 6.

**Table 6:** Li-ion battery pack specifications^37^.

Rated voltage	115 V
Rated capacity	4.4 Ah
Number of cells	32
Power	15 kW

### Ultracapacitor model

**Fig. 7:**
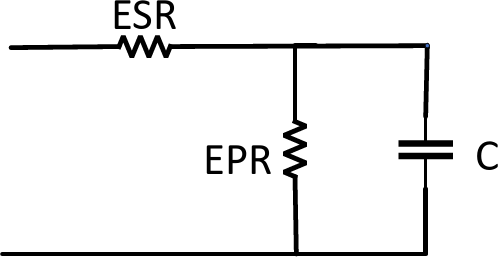
UC circuit diagram.

Ultracapacitors are electrical storage devices that present power density and high-power output. Their power density can be approximately 100 times higher than conventional capacitors and up to 10 times higher than batteries. High-power ultracapacitors may be charged and drained in 60 to 120 s on average, whereas high-power batteries take 10 to 15 min to charge and discharge. The classical equivalent circuit of the UC unit, shown in Fig. 7, consists of:

1. *C:* Capacitor.
2. *ESR:* An equivalent series resistance representing the charging and discharging resistance.
3. *EPR:* An equivalent parallel resistance representing the self-discharging losses.

The highest power demand for the cycle is 50 kW. Therefore, a 35 kW FC system and a 15 kW battery bank are appropriate for our design. The baseload power supplied by FC system ensures that the membrane is not subjected to sharp peak loads, thus increasing the lifetime of the FC system. This model is selected based on the ultracapacitor 48 V–165F produced by BMOD0165 P048. The following parameters were used to start the UC bank design, and UC specifications are presented in Table 7.

**Table 7:** Ultracapacitor module specifications.

Rated voltage	48 V
Rated capacitance	165 F
Number of cells	18
Maximum equivalent series resistance (ESR)	6.3 mΩ
Absolute maximum current	1900 A
Capacitance of individual cell	3000 F

Table 8 also shows the parameters of UC power calculation.

**Table 8:** UC power calculation.

dt	Time of discharge	10 s
Vmax	Maximum terminal voltage	432 V
Vmin	Minimum terminal voltage	48 V
Vw	Operating terminal voltage	240 V
P	Power rating	20 kW

The average current ($i_{avg}$) to be supplied by the UC bank is the average of the maximum ($i_{\max }$) and minimum ($i_{min}$) current demands from the UC bank.

9 $$i_{max} = \frac{P}{{V_{min} }} = \frac{20{,}000}{{48}} = 416.6\;{\text{A}}$$

10 $$i_{min} = \frac{P}{{V_{max} }} = \frac{20{,}000}{{432}} = 46.29\;{\text{A}}$$

11 $$i_{avg} = \frac{416.6 + 46.29}{2} = 231.44\;{\text{A}}$$

Total capacitance ($C_{{UC{ - }total}}$) is calculated in the following and where $n_{p}$ is the number of parallel capacitors and $n_{s}$ is the number of series capacitors and $R_{{UC{ - }total}}$ is the total resistance of the UC.

12 $$C_{UC - total} = C . \frac{{n_{p} }}{{n_{s} }}$$

13 $$C_{{UC{ - }total}} = 165 . \frac{2}{9} = 36.67 F$$

14 $$R_{{UC{ - }total}} = ESR . \frac{{n_{s} }}{{n_{p} }}$$

15 $$R_{{UC{ - }total}} = 6.3 *(10^{ - 3} )*\frac{2}{9} = 1.4*(10^{ - 3} )$$

The allowable voltage drop in the proposed design is expressed in Eq. (16) and total voltage drop $dV_{total}$ of the UC bank is the sum of the voltage drop due to the capacitive and resistive components^38^:

16 $$dV_{total} = Vw - Vmin = 240 - 48 = 192 V$$

17 $$\begin{gathered} dV_{total} = i_{avg} \cdot \frac{dt}{C} + R \cdot i_{avg} \hfill \\ dV_{total} = 231.44 \cdot \frac{10}{{36.67}} + 1.4*(10^{ - 3} )*231.44 = 63.5\;{\text{V}} \hfill \\ \end{gathered}$$

## Energy management strategy three RL loops and high level supervisory fuzzy control

The control strategy structure for HESS based on the Battery and UC/FC comprises two levels.

**Fig. 8:**
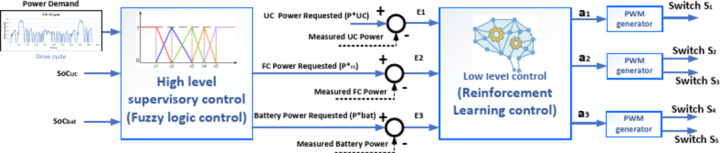
Overall solution procedure diagram of the proposed method.

The Energy Management strategy in EVs or FCEVs has a cascade with a two-layer control strategy and two different control layers. The first layer is a High-level supervisory control for power sharing, and the second is a low-level control strategy for switching power switches. These control layers aim to decrease fuel cell current stress and consider fuel cell ageing. A diagram of the overall solution procedure is drawn in Fig. 8 to illustrate the solution clearly.

### High-level supervisory control

**Fig. 9:**
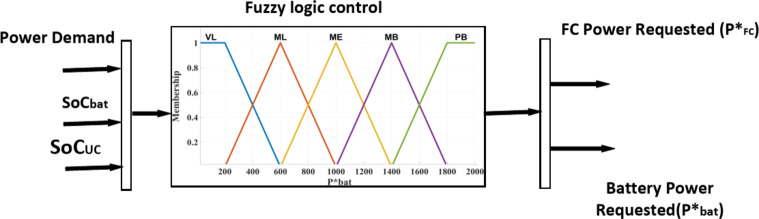
Schematic diagram of the high-level supervisory control for EMS.

These scops aim to decrease fuel cell current stress and consider fuel cell ageing. The proposed method will suggest a more robust, accurate, and precise system alongside uncertainties and disturbances in the EMS of FCHEVs. There is a main category rule-based in high-level supervisory control. An energy management system categorization is shown in Fig. 9. In the rule-based energy management system, power-sharing was determined by a set of predefined rules or look-up tables.

The energy management system uses the Mamdani inference structure with three input parameters and one output parameter that are vital to the energy management strategy.

1. The demand power is an important parameter for driving, which determines the output power of HESS.
2. Battery State of Charge (SoC_bat_) determines the endurance mileage of electric vehicles.
3. Ultracapacitor SoC (SoC_UC_) determines the instantaneous acceleration ability and climbing ability of electric vehicles.

**Fig. 10:**
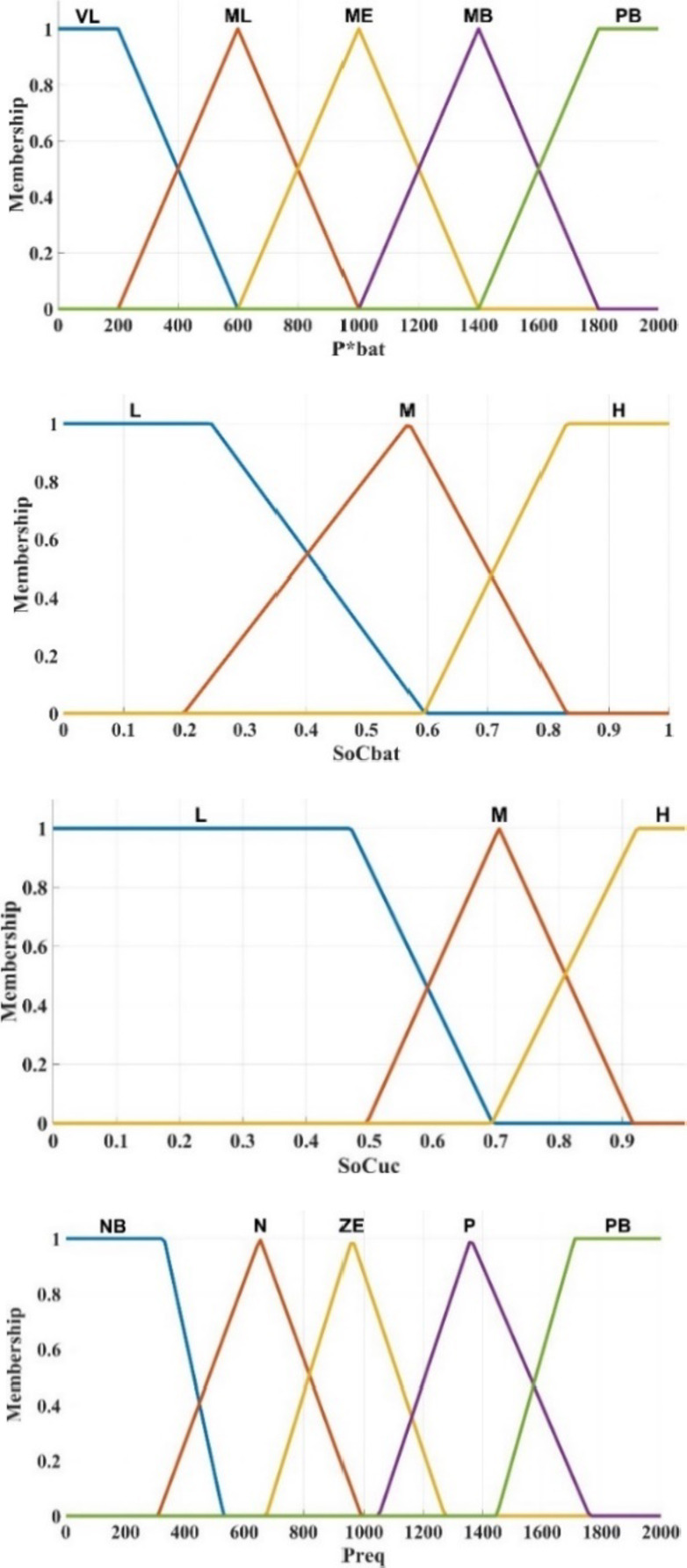
Fuzzy membership functions.

The fuzzy linguistic values (Table 9) and the input and output variables and membership functions are shown in Fig. 10. Therefore, the final fuzzy rule base, depending on the driver’s experience and expert experience, is shown in Table 10. The rules are categorized into 45 different cases based on the defined limits for SoC_bat_ and SoC_UC_ and P_demand_.

**Table 9:** Linguistic variables.

Variable	Fuzzy linguistic variables	Fuzzy range
P_demand_	NB, N, ZE, P, PB	[− 2000, 2000]
SoC_bat_	L, M, H	[0, 1]
SoC_uc_	L, M, H	[0, 1]
P*_bat_	VL, ML, ME, MB, PB	[0, 2000]

**Table 10:** Fuzzy rule bases.

P_demand_	SoC_uc_ (L) and SoC_bat_			SoCuc(M) and SoCbat			SoC_uc_(H)and SoC_bat_		
	L	M	H	L	M	H	L	M	H
NB	ML	VL	VL	ME	ML	VL	PB	PB	ME
N	ML	VL	VL	VL	ML	VL	ME	ML	PB
ZE	VL	VL	VL	VL	VL	VL	VL	VL	VL
P	ML	PB	PB	VL	ML	ME	VL	VL	ML
PB	ME	PB	PB	VL	ME	MB	VL	ML	ME

Input and output fuzzy variables based on linguistics are presented in the following:

**Load demand power (P**_**demand**_ **) as an input:**

NB (Negative Big), N (Negative), ZE (Zero), P (Positive), and PB (Positive Big).

**SoC as an input:**

L (Low), M (Medium), H (High).

Fuel cell and battery required power (P*_FC_ and P*_bat_ **):**

VL (Very Low), ML (Medium low), ME (Medium), MB (Medium Big), and PB (Positive Big).

### Low-level local control

Generally, the energy management problem in the form of RL is represented as a Markov decision process (MDP). The MDP is a mechanism that provides agents to learn and interact with their environment to achieve specific goals, known in the following:

18 $$MDP = \, (S,\;A,\;R,\;P)$$

Where *S* presents the state and the state vector are:

$$[s_{1} ,\;s_{2} , \ldots ,\;s_{n} ].$$

*n* means the dimension of the state space.

*A* shows the action set, which includes the sequence of action vectors [*a*_1_, *a*_2_, … , *a*_*k*_].

*k* is the dimension of the action space. *R* is the reward function, and *P* presents the dynamic transition probability of the environment.

**Fig. 11:**
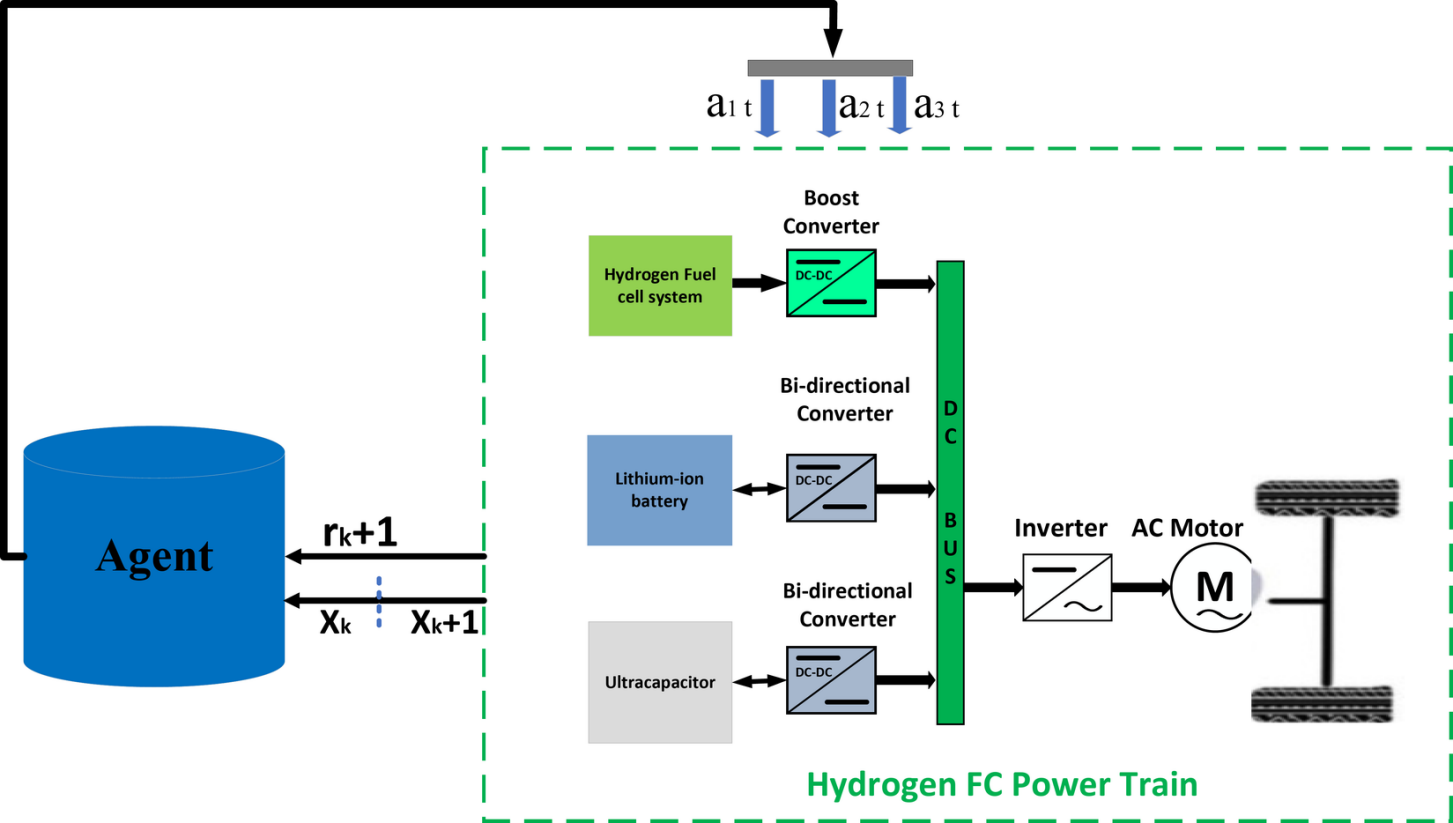
TD3-based low-level energy management for the fuel cell hybrid electric vehicle.

The TD3 agent is presented, and the training settings are explained. After training, the trained policy obtained from the TD3 agent is used in low-level control in the EMS. Figure 11 displays an overview of the TD3-based energy management for the fuel cell hybrid electric vehicle.

Regarding the current state *s* of the environment, the agent does an action *a* that follows a policy for the environment. The agent *r* takes a reward *r* for acting and a new state *s*’ from the environment. Based on this feedback, the agent updates the policy. The target is to find the policy *π* to maximize the action-value function.

The Q function is known as future discount rewards are given as follows:

19 $$Q(s,a) = {\mathbb{E}}\left[ {\mathop \sum \limits_{k = 0}^{T} \gamma^{k} r_{k} (s,a)} \right]$$

where *γ* is the discount factor.

#### TD3-based energy management strategy

**Fig. 12:**
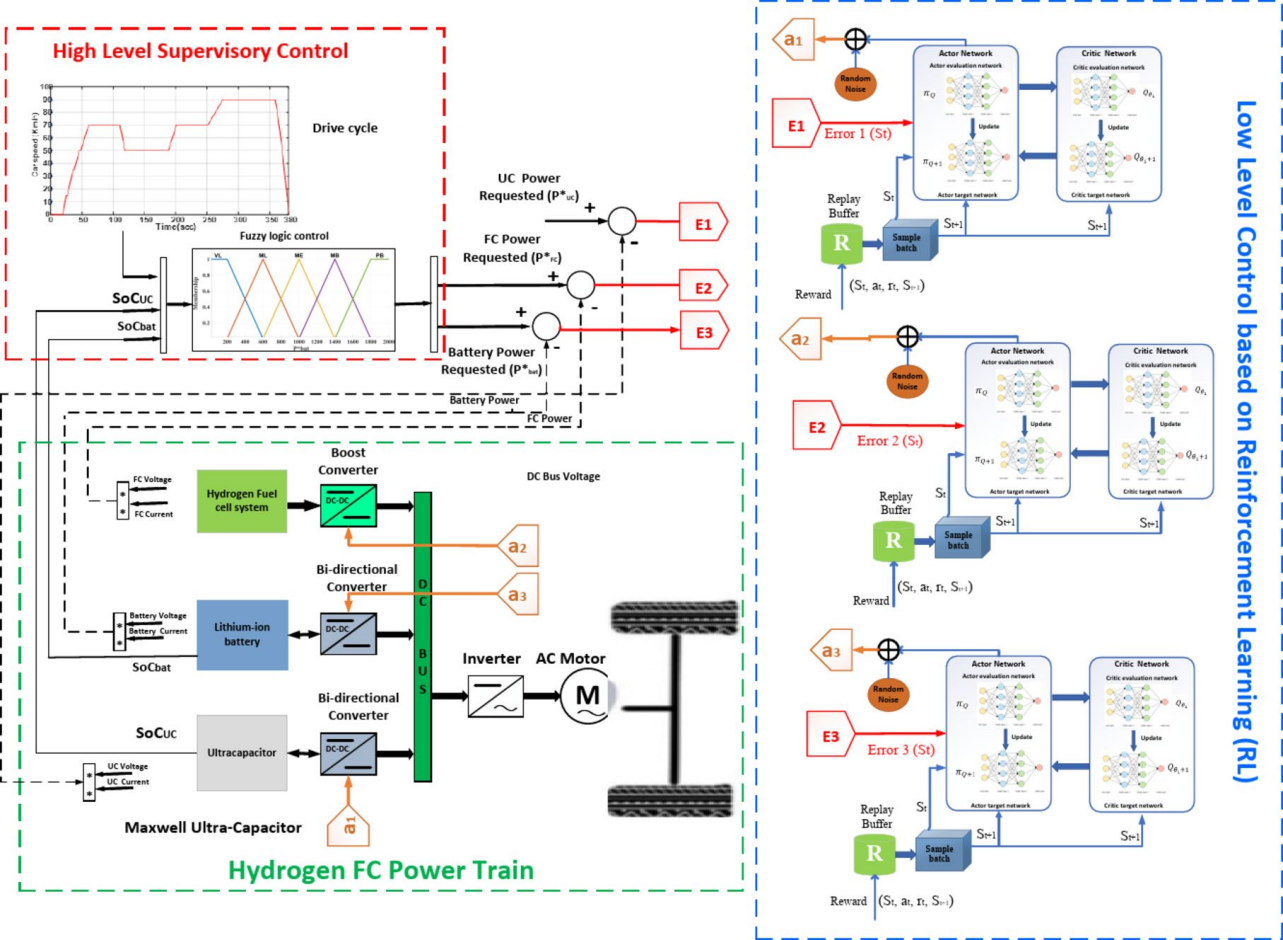
Layout of the proposed methodology-DRL configuration.

As in the DRL configuration shown in Fig. 12, the training environment includes the powertrain system with three TD3-based agents. The settings of agent actions, environment states, and rewards are critical to the interaction and learning of the agent.

#### TD3-based agent actions

The agent controls the output power of the battery/fuel cell/UC and regulate powers. Thus, the desired fuel cell power is a direct control input to the environment, where the fuel cell system is automatically controlled by a boost DC-DC converter to achieve the desired power output. The battery system in a closed-loop system is controlled by two Bi-directional DC-DC converters to achieve the desired power outputs are defined as:

20 $$a_{1} = \left[ {P_{fc} } \right];\;a_{2} = \left[ {P_{bat} } \right];\;a_{3} = \left[ {P_{UC} } \right]$$

#### Environment states

The agents need appropriate state information to manage the power precisely. Error 1 represents the error of the difference between the reference fuel cell power (P_fc_) and the measured fuel cell power. Error 2 mentions the error of the difference between the reference battery power (P_bat_) and the measured battery power. Where Error 3 shows the error of the difference between the reference UC power (P_UC_) and the measured UC power. Thus, the states of the environment are set to be:

$$s= [Error\; 1,\;\; Error\; 2,\;\; Error\; 3].$$

#### Rewards

The reward setting is crucial because the reward provides feedback to the agent on the effectiveness of the action and strongly affects the success of the training convergence.

The RL agent’s reward function (21) is the negative of the LQG criterion cost, as represented in Eq. (23). The RL agent maximizes this reward, thus minimizing the LQG cost.

In this work, the input weighting allows to maintain the desired power while minimizing the control effort *u*. The controllers in one closed-loop system are used the following criterion; also, there are three closed-loop structures in this research:

21 $${\text{Re}} wards = - \left( {\left( {P_{fc}^{*} \left( t \right) - P_{m} \left( t \right)} \right)^{2} + W*u^{2} \left( t \right)} \right)$$

where: *W*: Input weighting, *u(t)*: Control time-varying action signal.

22 $$J = \mathop {\lim }\limits_{T \to \infty } E\left( {\frac{1}{T}\mathop \smallint \limits_{T0}^{Tf} \left( {\left( {P_{fc}^{*} \left( t \right) - P_{m} \left( t \right)} \right)^{2} + W*u^{2} \left( t \right)} \right) \cdot dt} \right)$$

#### Training settings

For the proposed TD3-based energy management system, a training procedure should be performed before testing. The settings for training the TD3-agent are summarized in Table 11.

**Table 11:** TD3 agent settings for training.

Networks and parameters	Settings and values
Actor networks	Fully connected layers 400/200/100 units
Actor learning rate	1e−3
Critic networks	Fully connected layers 400/200/100 units
Critic learning rate	1e−3
Optimizer type Adam	Adam
Batch size	128
Delayed policy update iterations	10
Discount factor	0.99
Exploration policy	*Variance* = 0.1

## Evaluations and results

In this section, the proposed Deep reinforcement learning-based energy management system (DRL-EMS) controller has been verified using varying load conditions under the European Extra Urban Driving Cycle (EUDC) by generating reference current for FC, battery and UC by controlling three converters.

The entire system of FCEV and the proposed algorithms are simulated in MATLAB/Simulink^®^(2023b) software, the sampling size is 1e−4, solver type is the variable step of ode23tb (stiff/TR-BDF2).

A proposed controller is designed to share the energy between the battery and ultracapacitor, and it is proposed to reduce peak power and prolong the battery life to meet SoC of the battery and ultracapacitor constraint by optimizing the parameters of the fuzzy membership functions.

**EUDC test**

**Fig. 13:**
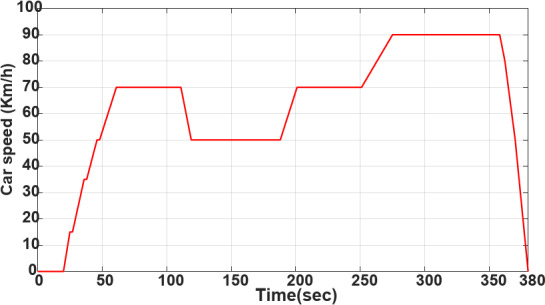
Varying EUDC speed profile of the vehicle.

**Fig. 14:**
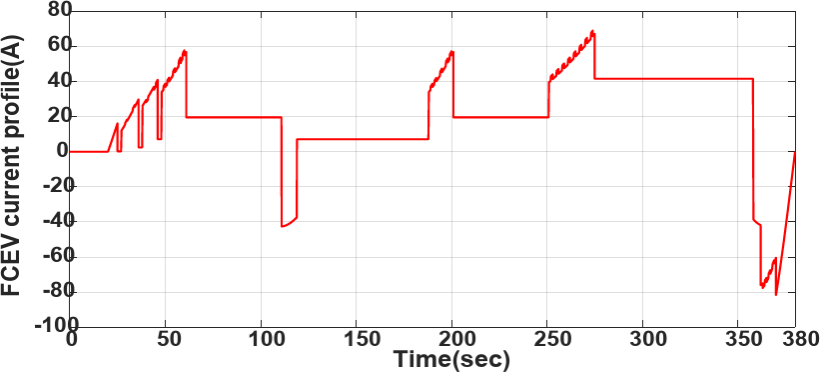
Varying load current profile used for EV.

Figures 13 and 14 demonstrate the vehicle’s EUDC speed profile and varying load current profile. The vehicle can achieve a maximum speed of 90 km/h. Figure 14 displays the current profile under varying load current demand from *t* = 0 s to *t* = 380 s.

### Comparison of power sharing/current profile tracking in different strategies

**Fig. 15:**
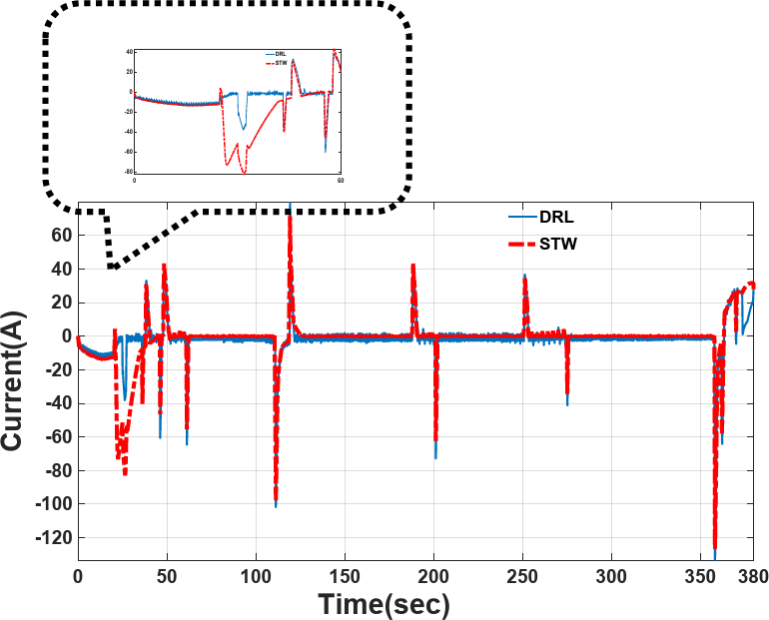
Current tracking error of DRL-F and STW-F.

**Fig. 16:**
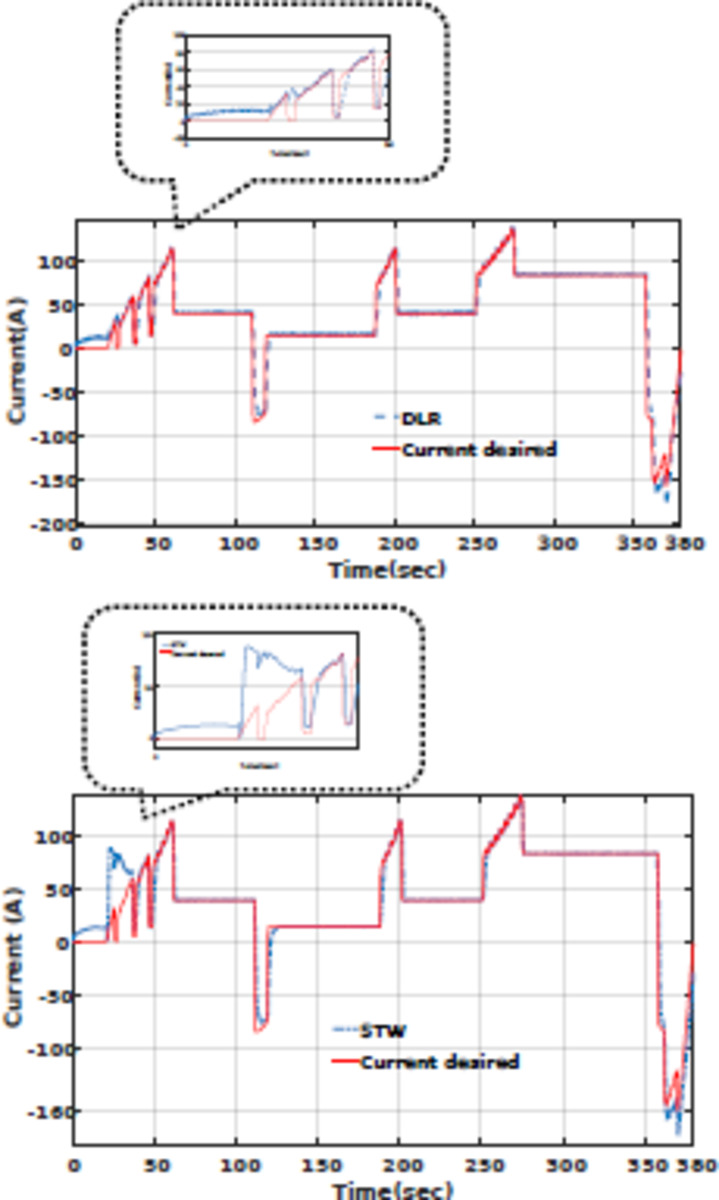
Load current tracking by DRL-F and STW-F used for EV.

The power-sharing of proposed Deep Reinforcement Learning (DRL), and Fuzzy logic supervisory control (DRL-F) is compared to the Super-Twisting algorithm (STW) and Fuzzy logic supervisory control (STW-F) under EUDC driving cycle conditions are shown in Figs. 15, 16, respectively. Figure 14 demonstrates the current output profile for FCHEV and its reference.

Super twisting algorithm is a type of sliding mode control which is known as second-order sliding mode control that is supposed by Levant et al.^39^ and used in several applications in^40,41^ that can be written as follows:

23 $$\begin{gathered} u_{STW} = u_{a} + u_{b} \hfill \\ u_{a} = - \lambda \left| s \right|^{\frac{1}{2}} sign(s) \hfill \\ \dot{u}_{b} = - W sign(s) \hfill \\ \end{gathered}$$

A sliding surface $\left( s \right)$ is defined as the error between the real load current and the reference current.

Controller coefficients such as $\lambda$ = 300, and ${\text{W}}$ = 100 are calculated to meet the desired objectives such as reducing the chattering.

In Fig. 15, the desired current of FCHEV can be observed from *t* = 0 s to *t* = 380 s as well as the tracking of the desired current by the proposed controller DRL-F can be seen and compared to STW-F. The current tracking error is shown in Fig. 15 and is very close to zero. However, DRL is direct to zero faster than the STW method. It is obvious in the Zoom picture.

It can be observed that the DRL-F controller tracks the desired reference more accurately and faster, and the errors approach zero, which is a requirement for the system. Some undershoots and overshoots are shown due to load variations, but the overall performance of the proposed algorithms is satisfactory.

Table 12 shows the comparison between two strategies (DRL-F, STW-F) under the EUDC driving cycle for the Root Mean Square Error (RMSE), Mean Square error (MSE), and Mean error. Error is defined in the following and it is clear for better operation of DRL-F strategy rather than STW-F.

$$Error = Desired \, \;output\; \, current{-}Measured \, \;output\; \, current$$

**Table 12:** Comparison results for current tracking error.

Driving test	Control strategy	Mean error	Root mean square error	Mean square error
EUDC	STW-F	− 1.7077	14.3475	205.8511
EUDC	DRL-F	− 1.5658	11.3289	128.3440

Simulation results show that, compared with STW-F EMS, the proposed strategy achieves less Mean Error of power tracking 0.1419 with 8.31%. Less Mean Square Error for DRL-F of 77.59 with 37.65% compared to the STW-F algorithm.

Results in Table 12 indicate that the proposed model of DRL-F can ensure the RMSE reduction of 3.0186 for 21.05% compared to the STW-F.

The power-sharing of the proposed DRL-F, and STW-F under EUDC driving cycle conditions are shown in Fig. 16. However, the DRL-F proposed method shows accurate tracking of the desired current. However, STW-F, as a robust controller, has lower tracking accuracy in the first times of simulation until below 50 s of power demand. Load current tracking in Fig. 16 shows that lower tracking accuracy is between 25 to 50 s of STW-F used for EV with high peak and without any power tracking, which is clear in this picture’s zoom.

**Fig. 17:**
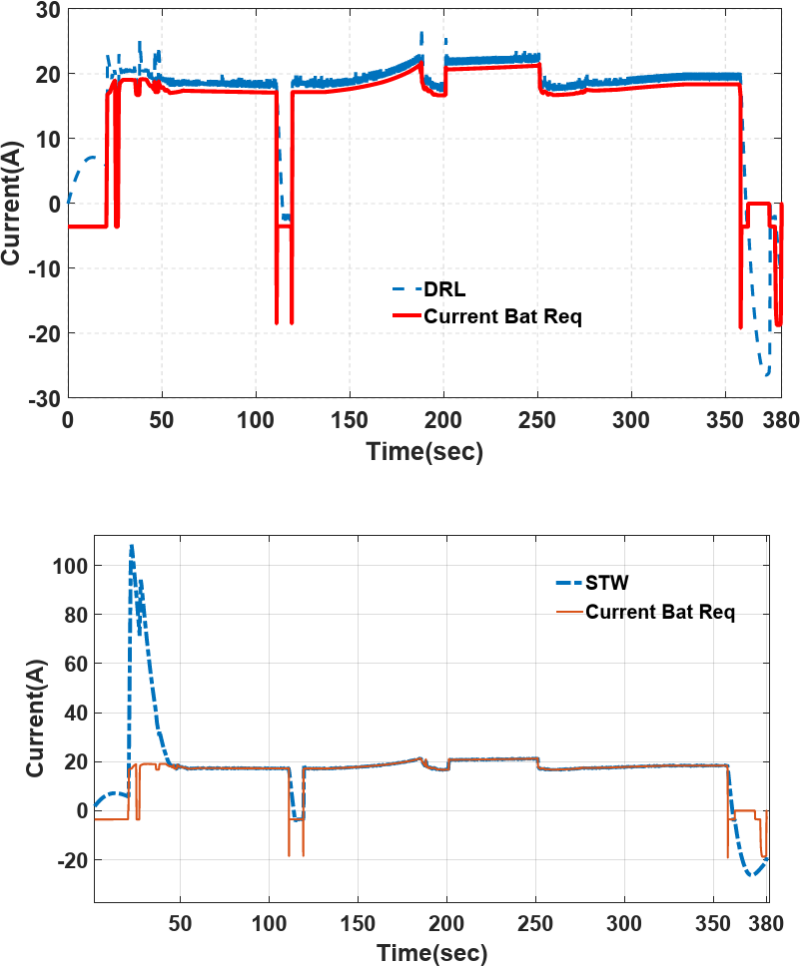
Varying load current profile used for EV.

The DRL-F in Fig. 17 displays that the desired Li-ion battery power can allocate less power than STW-F. STW-F has a big overshoot and peak power in the first times of simulation until below 50 s. When the Battery Current is less than zero (Battery Current < 0), the battery goes into the charging mode also, it is in the discharging mode for Battery Current > 0, depending on the SoC of the battery and UC.

**Fig. 18:**
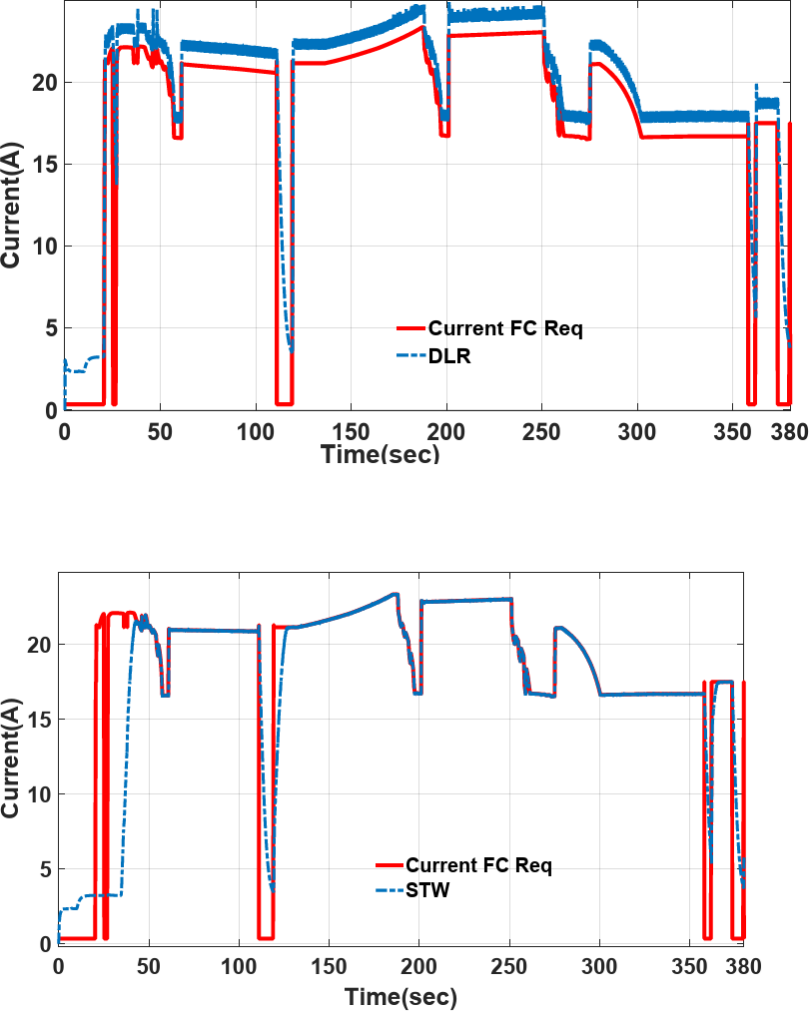
Tracking of the PEMFC current desired by DRL and STW-F.

Also, Fig. 18 shows the tracking of the PEM fuel cell current desired by DRL and STW-F, in which DRL-F is more precise and faster current tracking rather than STW-F. Desired PEMFC current can be allocated faster in the first second than STW-F. STW-F takes time to achieve the reference fuel cell power. It is shown in the figure that tracking the desired PEMFC current by STW-F in below 50 s does not happen, and there is no tracking below 50 s, which increases inaccuracy and errors in power tracking.

From *t* = 120s to *t* = 130s the load current is negative (Fig. 14), according to the high-level supervisory control of fuzzy and fuzzy rules presented in Table 10, when the current is negative and FCHEV is on regenerative braking mode, and the SoC of battery is low with the SoC of UC is *low*, the FC is low power or idle mode and battery and UC is charged. It is presented in Figs. 16, 17, and 18 from *t* = 120 s to *t* = 130 s. This confirmed the results obtained, and the effectiveness of the Proposed algorithms can be observed.

Another aspect of the results noted is that less use of batteries in the power-sharing process causes an increase in fuel cell lifetime and battery lifetime, a decrease in energy cost, and a reduction in electricity cost for battery charging. Therefore, both DRL-F and STW-F under EUDC can share the demand power by taking advantage of Ultracapacitors in the HESS. High-level supervisory control and DRL low-level control are proposed for transient power-sharing provided by UC and flat-level power-sharing and low-stress loads delivered by fuel cell and battery to reduce current stress on fuel cell and prevent fuel cell damage and ageing.

**Fig. 19:**
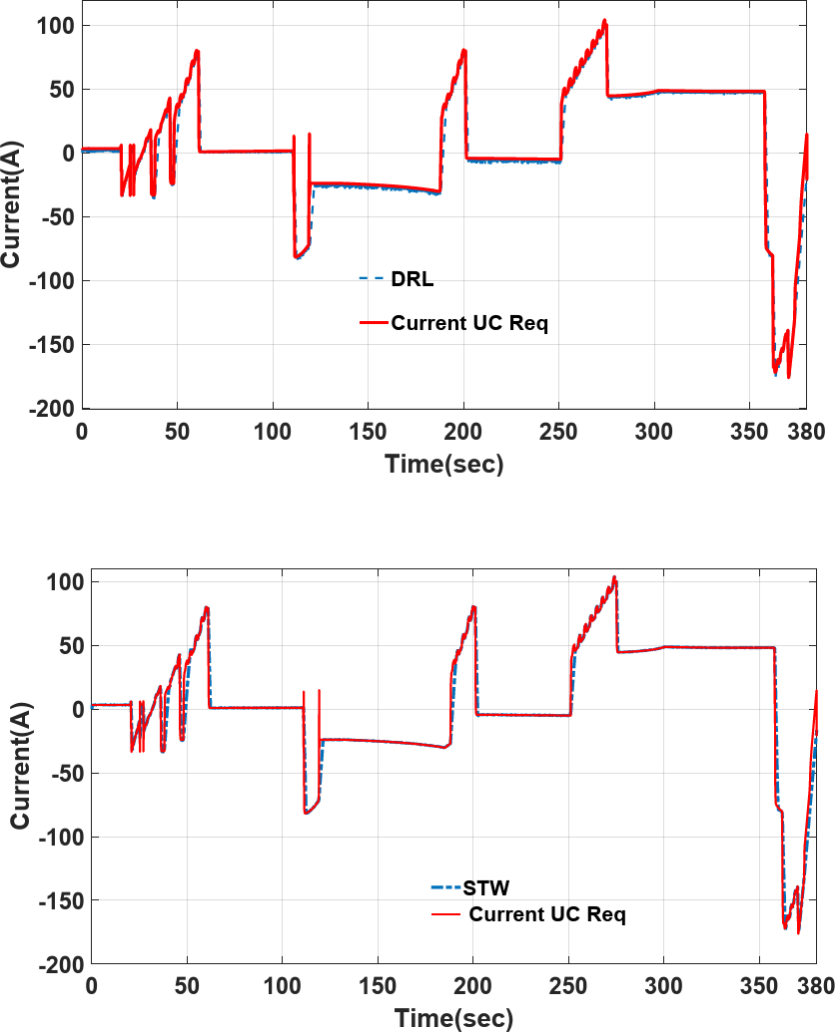
Transient current delivered by UC on load current profile.

The UC power-sharing of the proposed DRL-F under EUDC driving cycle condition is shown in Fig. 19 and is compared with STW-F, and it is clear that UC delivers transient power.

It can be noted that when the load current is getting high peaks and transient load currents (from t = 20 s to 60 s, 187 s to 200 s, and 250 s to 275 s) UC start discharging to meet the load requirements. There is a reduction in fuel cell and battery usage, the ultracapacitor operates faster than FC and battery throughout the driving cycle; some benefits described as follows:

- Increasing accuracy of power tracking
- The lithium battery’s output and peak current reduction, thus gaining fuel cell and battery lifetime.
- Flat-power sharing is delivered by PEMFC, and battery Fuel cell ageing, and destruction are prevented.
- Regenerative braking energy across the driving saved in UC in a short time and prevents energy consumption.
- Facilitate ultracapacitor’s charging through regenerative braking mechanisms.

**Fig. 20:**
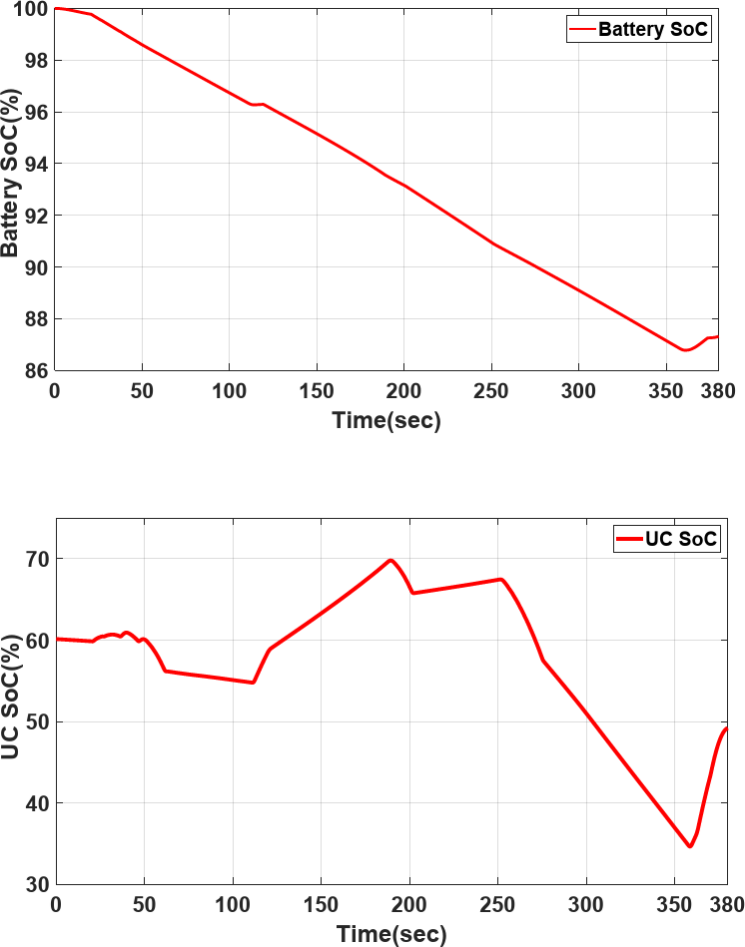
Variation range of the Li-ion battery SoC_bat_ and Ultracapacitor SoC_UC_ under EUDC profile.

Figure 20 shows the variation range of the Li-ion battery SoC_bat_ and Ultracapacitor SoC_UC_ optimized by DRL-F. Meanwhile, the terminal SoC_bat_ and U SoC_UC_ show that the UC charging numbers are higher than the battery charging. It mentions that overall power sharing and transient load current are expanding battery lifetime.

**Fig. 21:**
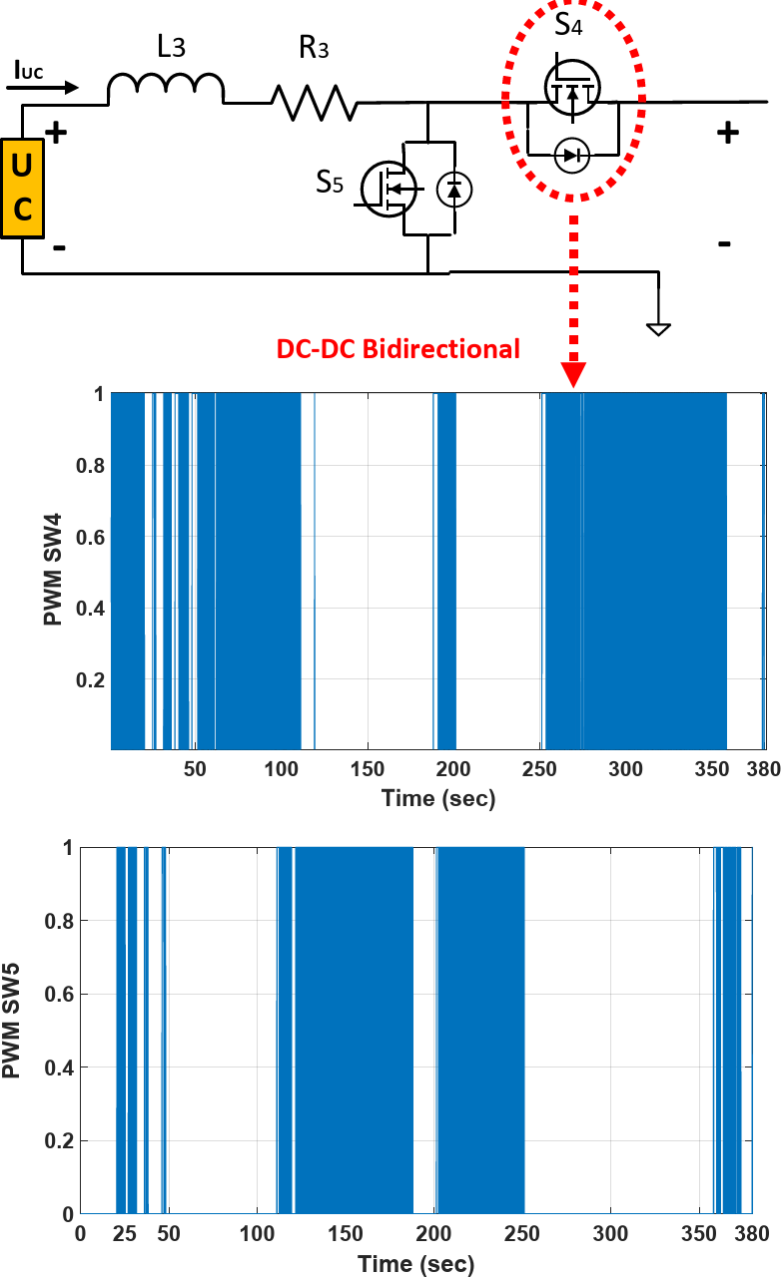
PWM switching’s of SW_4_ and SW_5_ of bi-directional converter on FCHEV.

PWM switching of SW_4_ and SW_5_ of UC bi-directional converter on FCHEV is shown in Fig. 21, and PWM signals in buck mode and boost modes are shown and signals are placed under each other, and it is obvious that signals cover each other in each mode (Buck-Boost) as a bi-directional converter.

## Conclusion

In this research, hydrogen-based electric vehicle-PEMFC electric vehicles have been studied. PEM fuel cell was a main source and a hybrid energy storage system, including a battery and an Ultra-capacitor. The combination of reinforcement learning algorithms in low-level control loops and high-level supervisory control based on fuzzy logic load sharing was designed for FCHEV.

A powertrain system for FCHEV was implemented with three DC-DC converters, two bi-directional and one boost converter. A hierarchical energy management framework was employed in a two-layer control strategy. Three loop control strategies based on reinforcement learning were designed to improve FC and battery power-sharing tracking in the low-level layer control strategy.

The DRL-F energy management system has been designed to address various performance metrics of energy consumption, fuel cell lifetime, and battery lifetime, as well as the reduction of transient and peak current on PEMFC and Li-ion batteries. Deep reinforcement learning control was used, and three DRL-PID controllers were designed using the hierarchical energy optimization control architecture. Finally, the performance of the obtained controller was compared with a sliding mode super-twisting algorithm. The proposed method suggested a more robust, accurate and precise system alongside uncertainties and disturbances in EMS of FCHEVs with an advanced learning method.

This paper proposed a novel energy management method to improve the lifetime of fuel cells and batteries because the UC is faced with transient and peak current demands, and it can save batteries and fuel cells for any peak transient demands. In future research, we will develop a new method, deep learning, to measure the lifetime of fuel cells. The lifetime of fuel cells will be measured online using AI approaches.

## Data Availability

Some or all data, models, or codes that support the findings of this study are available from the corresponding author upon reasonable request.

## Abbreviations

HESS: Hybrid energy storage system
EMS: Energy management strategy
UC: Ultracapacitor
DRL: Deep Reinforcement Learning
PID-RL: PID-Reinforcement Learning
DDPG-TD3: Deep Deterministic Policy Gradient with Twin Delayed
EUDC: Extra-urban driving cycle
SoC_UC_: Ultracapacitor state of charge
SoC_bat_: Battery state of charge
Voc: Open-circuit voltage
$R_{series }$: DC battery series resistance
$$I_{Bat}$$: Battery current
$$I_{UC}$$: Ultracapacitor current
$R_{UC }$: Ultracapacitor internal resistance
$$C_{UC}$$: Ultracapacitor capacitance
$V_{UC}^{oc}$: Ultracapacitor open-circuit voltage
$$I_{Bat}$$: Battery current
RMSE: Root-mean-square error
$$C_{p}$$: Polarisation capacitance
$R_{p}$: Polarisation internal resistance
$V_{Bat}$: Battery terminal voltage
$V_{R}$: Battery partial voltage of resistance–capacitance network
$V_{ocv}^{Bat}$: Battery open-circuit voltage
$R_{series }$: DC internal resistance
$P_{req}$: Demand for power
$$V_{cp}$$: Capacitor voltage
